# Briarenols C–E, New Polyoxygenated Briaranes from the Octocoral *Briareum excavatum*

**DOI:** 10.3390/molecules22030475

**Published:** 2017-03-17

**Authors:** Nan-Fu Chen, Yin-Di Su, Tsong-Long Hwang, Zuo-Jian Liao, Kuan-Hao Tsui, Zhi-Hong Wen, Yang-Chang Wu, Ping-Jyun Sung

**Affiliations:** 1 Division of Neurosurgery, Department of Surgery, Kaohsiung Armed Forces General Hospital, Kaohsiung 802, Taiwan; chen06688@gmail.com; 2 Department of Neurological Surgery, Tri-Service General Hospital, National Defense Medical Center, Taipei 114, Taiwan; 3 National Museum of Marine Biology and Aquarium, Pingtung 944, Taiwan; gobetter04@gmail.tw (Y.-D.S.); liao771107@gmail.com (Z.-J.L.); 4 Graduate Institute of Natural Products, College of Medicine and Chinese Herbal Medicine Research Team, Healthy Aging Research Center, Chang Gung University, Taoyuan 333, Taiwan; htl@mail.cgu.edu.tw; 5 Research Center for Chinese Herbal Medicine, Research Center for Food and Cosmetic Safety and Graduate Institute of Health Industry Technology, College of Human Ecology, Chang Gung University of Science and Technology, Taoyuan 333, Taiwan; 6 Department of Anesthesiology, Chang Gung Memorial Hospital, Taoyuan 333, Taiwan; 7 Doctoral Degree Program in Marine Biotechnology, National Sun Yat-Sen University and Academia Sinica, Kaohsiung 804, Taiwan; wzh@mail.nsysu.edu.tw; 8 Department of Obstetrics and Gynecology, Kaohsiung Veterans General Hospital, Kaohsiung 813, Taiwan; khtsui60@gmail.com; 9 Department of Obstetrics and Gynecology and Institute of Clinical Medicine, National Yang-Ming University, Taipei 112, Taiwan; 10 Department of Biological Science, National Sun Yat-Sen University, Kaohsiung 804, Taiwan; 11 Department of Pharmacy and Master Program, College of Pharmacy and Health Care, Tajen University, Pingtung 907, Taiwan; 12 Department of Marine Biotechnology and Resources, National Sun Yat-Sen University, Kaohsiung 804, Taiwan; 13 Graduate Institute of Natural Products, Kaohsiung Medical University, Kaohsiung 807, Taiwan; 14 Research Center for Natural Products and Drug Development, Kaohsiung Medical University, Kaohsiung 807, Taiwan; 15 Department of Medical Research, Kaohsiung Medical University Hospital, Kaohsiung 807, Taiwan; 16 Graduate Institute of Marine Biology, National Dong Hwa University, Pingtung 944, Taiwan; 17 Chinese Medicine Research and Development Center, China Medical University Hospital, Taichung 404, Taiwan

**Keywords:** *Briareum excavatum*, octocoral, briarane, briarenol, anti-inflammatory, elastase, iNOS

## Abstract

Three new polyoxygenated briarane diterpenoids, briarenols C–E (**1**–**3**), were isolated from the octocoral *Briareum excavatum*. The structures of briaranes **1**–**3** were elucidated by interpretation of spectroscopic data, and the methylenecyclohexane ring in **1** was found to exist in a twisted boat conformation. Briarenol D (**2**) displayed an inhibitory effect on the release of elastase by human neutrophils with an IC_50_ value of 4.65 μM. Briarenol E (**3**) was found to inhibit the protein expression of pro-inflammatory inducible nitric oxide synthase (iNOS) in a murine macrophage-like cell line, RAW 264.7, stimulated with lipopolysaccharides (LPS).

## 1. Introduction

Over the past 40 years, over 600 diterpenoids possessing the briarane carbon skeleton, most of which were found to contain a γ-lactone moiety in a bicyclo[8.4.0] system, have been isolated from marine coelenterates, mainly from the octocorals [1,2,3,4,5,6]. Increasing interest is being paid to these briaranes, not only due to their complex structures, but also owing to their interesting diverse bioactivities, such as anti-inflammatory activity [7,8,9]. In a continuing survey of Taiwanese marine invertebrates with promising novel briaranes, the octocoral *Briareum excavatum* (family Briareidae) was investigated. In this paper, we report the isolation, structure determination, and bioactivity of three new polyoxygenated briaranes, briarenols C–E (**1**–**3**), following further study of *B. excavatum* (Figure 1 and Supplementary Figures S1–S21).

## 2. Results and Discussion

Briarenol C (**1**) was obtained as a white amorphous powder. The high-resolution electrospray ionization mass spectrum (HRESIMS) showed a signal at *m*/*z* 545.19930 (calcd. for C_26_H_34_O_11_ + Na, 545.19933), and therefore the molecular formula of **1** was determined to be C_26_H_34_O_11_ (10° of unsaturation degrees). Analysis of the IR spectra of **1** showed absorptions at 3428, 1772, and 1734 cm^−1^, indicating that the structure of **1** consisted of hydroxy, γ-lactone, and ester groups. Based on the results of the ^13^C-NMR and distortionless enhancement by polar transfer (DEPT) spectra (Table 1), the presence of a trisubstituted olefin and an exocyclic carbon-carbon double bond was deduced from the signals of four carbons at δ_C_ 150.6 (C-11), 144.8 (C-5), 123.4 (CH-6), and 109.3 (CH_2_-20); this was further supported by three olefin proton signals at δ_H_ 5.59 (1H, d, *J* = 10.0 Hz, H-6), 5.13 (1H, d, *J* = 1.2 Hz, H-20a), and 5.03 (1H, s, H-20b) in the ^1^H-NMR spectrum of **1** (Table 1). Four carbonyl resonances at δ_C_ 171.9 (C-19), 170.6 (an ester carbonyl), and 170.3 (2 × ester carbonyls) confirmed the presence of a γ-lactone and three esters in **1**; three acetyl methyls (δ_H_ 2.04, 2.01, 1.91, each 3H × s) were also observed. According to the overall unsaturation data, **1** was concluded to be a diterpenoid molecule possessing four rings. The presence of a tetrasubstituted epoxide that contained a methyl substituent was revealed by the signals of two oxygenated quaternary carbons at δ_C_ 71.4 (C-8) and 60.6 (C-17), and was further confirmed by the proton signal of a methyl singlet resonating at δ_H_ 1.53 (3H, s, H_3_-18).

The ^1^H-NMR coupling information obtained from the ^1^H-^1^H correlation spectroscopy (COSY) spectrum of **1** indicated the existence of H-2/H_2_-3/H-4, H-6/H-7, H-9/H-10, H-12/H_2_-13/H-14, and H-6/H_3_-16 (by allylic coupling) units (Table 1), which were established with the assistance of a heteronuclear multiple bond coherence (HMBC) experiment. The HMBC correlations between protons and quaternary carbons of **1**, such as H-2, H-3β, H-10, H-13α, H-14, H_3_-15/C-1; H-3α, H-7, H_3_-16/C-5; H-6, H_3_-18/C-8; H-10, H-13β/C-11; H-9, H_3_-18/C-17; and H_3_-18/C-19, allowed clarification of the carbon skeleton (Table 1). An exocyclic double bond at C-11 was confirmed by the HMBC correlations between H_2_-20/C-10, -12. The presence of a methyl group at C-5 was concluded based on the results of allylic coupling between H-6/H_3_-16 in the ^1^H-^1^H COSY spectrum and by HMBC correlations between H_3_-16/C-4, -5, -6 and H-4/C-16. The junction C-15 methyl group was positioned at C-1, as HMBC correlations were found between H-2/C-15; H-10/C-15; and H_3_-15/C-1, -2, -10, -14. Two acetoxy groups were found to be attached at C-2 and C-14, respectively, based on the presence of HMBC correlations between H-2 (δ_H_ 4.80), H-14 (δ_H_ 4.74) and the acetate carbonyls at δ_C_ 170.6 and 170.3. Therefore, the remaining acetoxy and two hydroxy groups were inferred to be located at C-4, C-9, and C-12, respectively, as suggested by analysis of ^1^H-^1^H COSY correlations and characteristic NMR signals, even though no HMBC correlation was observed between H-4 (δ_H_ 5.19) and the acetate carbonyl.

The proton chemical shifts of the briarane derivatives contained an 11,20-exocyclic carbon- carbon double bond. The difference between the two olefin protons (H-20a/b) was smaller than 0.2 ppm, whereas the cyclohexane rings exhibited a twisted boat conformation [10]. Owing to the chemical shifts of the C-20 methylene protons (δ_H_ 5.13 and 5.03), the configuration of the methylenecyclohexane ring in **1** was concluded to exist in a twisted boat conformation. The configuration of **1** was elucidated from the interactions observed in a nuclear Overhauser effect spectroscopy (NOESY) experiment (Figure 2) and from vicinal proton coupling constant analysis. In the NOESY experiment, the correlations of H-10 with H-2 and H-9, but not with H_3_-15, demonstrated that these protons (H-2, H-9, and H-10) were located on the same face of the molecule, and could be assigned as α protons, as Me-15 was a β-substituent at C-1. H-14 was found to exhibit a correlation with H_3_-15, but not with H-10, indicating that this proton was of a β-orientation at C-14. H-12 was found to be correlated with H_3_-15, but not with H-10; one proton of the C-20 methylene (δ_H_ 5.03, H-20b) was correlated with H-9 and H-10, suggesting that the C-12 hydroxy group was α-oriented. This was further supported by the fact that the methylenecyclohexane ring in **1** existed in a twisted boat conformation. The Z-configuration of the C-5/6 double bond was elucidated from the correlation between the C-6 olefin proton (δ_H_ 5.59) and the C-16 vinyl methyl (δ_H_ 2.20). One proton of the C-3 methylene (δ_H_ 3.15) was correlated with H_3_-15, but not with H-2, and it was therefore assigned as an H-3β proton. H-7 showed a correlation with H-3β, but not with H-6, and a large coupling constant was detected between H-7 and H-6 (*J* = 10.0 Hz), indicating that the dihedral angle between H-6 and H-7 was approximately 180°, and H-7 was β-oriented. Due to H-4 exhibiting a NOE interaction with H_3_-16, and a doublet coupling having been identified between H-4 and the C-3 methylene protons (*J* = 12.8, 6.0 Hz), the acetoxy group at C-4 was identified as being β-oriented. H-9 was found to be correlated with H-10, H_3_-18, and H-20b, and from consideration of molecular models, H-9 was found to be reasonably close to H-10, H_3_-18, and H-20b; therefore, H-9 could be placed on the α face in **1**, and H_3_-18 was β-oriented in the γ-lactone moiety.

Since 1977, when the first briarane-type diterpenoid, briarein A, was isolated from the Caribbean octocoral *Briareum asbestinum* [11], all naturally derived briarane-based diterpenoids prepared from octocorals belonging to the genus *Briareum* have been found to possess a C-15 methyl group at C-1 trans to H-10, and these two groups were proven to be β- and α-oriented, respectively. Based on biosynthetic derivation, the absolute configurations of the chiral carbons of **1** were assigned as 1*R*, 2*S*, 4*R*, 7*S*, 8*R*, 9*S*, 10*S*, 12*R*, 14*S*, and 17*R*.

Briarenol D (**2**) had the same molecular formula as **1**, C_26_H_34_O_11_, as determined by HRESIMS at *m*/*z* 545.19950 (calcd. for C_26_H_34_O_11_ + Na, 545.19933) with 10 degrees of unsaturation, indicating that compounds **1** and **2** were isomers. By detailed ^1^H, ^13^C, and 2D NMR spectroscopic analysis (Table 1 and Table 2), compound **2** was found to have the same substituents as **1** (three acetoxy and two hydroxy groups). On the basis of the ^1^H-^1^H COSY spectrum of **2** (Table 2), it was possible to establish the sequences of the protons attached to the carbon skeleton of **2**. Furthermore, a hydroxy proton signal at δ_H_ 3.00 (1H, d, *J* = 4.8 Hz) was correlated in the ^1^H-^1^H COSY spectrum with H-9 (δ_H_ 4.35, 1H, br s), indicating that this hydroxy group was positioned at C-9. The results of the HMBC correlation analysis of **2** confirmed the positions of the acetoxy groups at C-2 and C-4 by the connectivities between the oxymethine protons at δ_H_ 5.01 (H-2), 5.08 (H-4) and δ_C_ 170.4, 170.3 (2 × acetate carbonyls), respectively. Therefore, the remaining hydroxy and acetoxy groups were positioned at C-12 and C-14, respectively, as indicated by analysis of ^1^H-^1^H COSY correlations and characteristic NMR signals analysis, even though no HMBC correlation was observed between H-14 (δ_H_ 4.76) and the acetate carbonyl. The stereochemistry of the stereogenic centers in the 10-membered ring (C-1, C-2, C-4, C-7, C-8, C-9, and C-10) and the γ-lactone moiety (C-17) of **2** was confirmed to be the same as that of **1** by comparison of the proton shifts, coupling constants, and NOESY correlations. The hydroxy and acetoxy groups at C-12 and C-14 were assigned β- and α-configurations, primarily due to NOESY correlations between H-10/H-12 and H-14/H_3_-15, respectively. Thus, the methylenecyclohexane ring in **2** existed in a chair conformation, and the stereogenic centers of **2** were assigned as 1*R*, 2*S*, 4*R*, 7*S*, 8*R*, 9*S*, 10*S*, 12*S*, 14*S*, and 17*R*.

**Figure 1:**
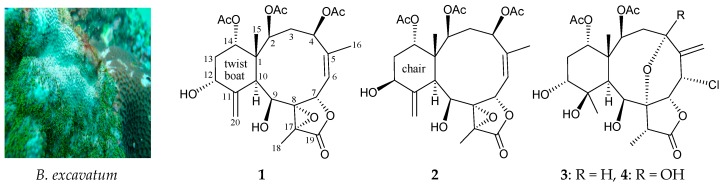
The octocoral *Briareum excavatum* and the structures of briarenols C–E (**1**–**3**) and briarenolide ZI (**4**).

**Figure 2:**
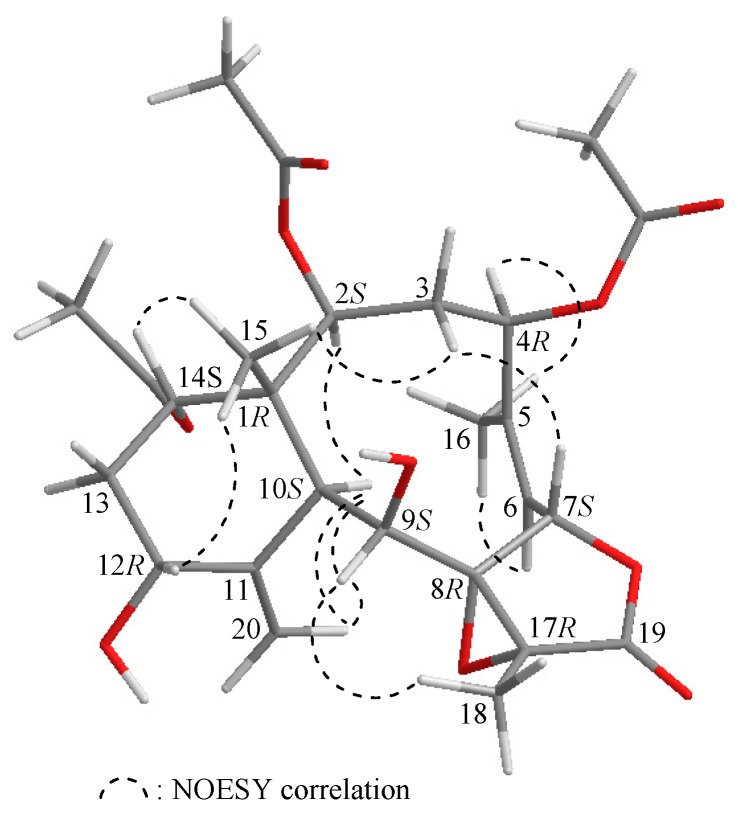
Selected protons with key NOESY correlations of **1**.

Briarane **3** (briarenol E), with a molecular formula of C_24_H_33_ClO_10_ (on the basis of HRESIMS; *m*/*z* 539.16555, calcd. for C_24_H_33_ClO_10_ + Na, 539.16545), was recognized as a 6-chlorinated briarane diterpenoid closely related to a known briarane, briarenolide ZI (**4**) [12] (Figure 1), based on data obtained by 1D and 2D NMR analysis (Table 3). Briaranes **3** and **4** had identical substituents: secondary acetoxy groups at C-2 and C-14; an exocyclic methylene at C-5; a chloride atom at C-6; secondary hydroxy groups at C-9 and C-12; and a tertiary hydroxy group at C-11. In addition, they also had an ether bridge between C-4/8 in common. While briarane **4** was found to contain a tertiary hydroxy group at C-4 of the pyran ring, **3** had a hydrogen atom at that position. The ^1^H- and ^13^C-NMR data assignments of **3** were made in comparison with those of **4**. The ^1^H-^1^H COSY and HMBC correlations observed fully supported the derived locations of the functional groups. Briarane **3** was assigned as having a structure with the same stereochemistry as that of **4**, because for the stereogenic centers that **3** has in common with **4**, the ^1^H- and ^13^C-NMR chemical shifts and proton coupling constants matched well. Based on the above findings, the stereogenic centers of **4** were assigned as 1*R*, 2*S*, 4*S*, 6*S*, 7*R*, 8*R*, 9*S*, 10*S*, 11*R*, 12*R*, 14*S* and 17*R*. Thus, this compound was found to be the 4-dehydroxy derivative of briarenolide ZI (**4**) [12].

In in vitro anti-inflammatory activity assays, it was found that briarane **2** showed a selective inhibitory effect on the release of elastase with an IC_50_ value of 4.65 μM, by human neutrophils (Table 4). Briarane **1** was found to be inactive on the above two anti-inflammatory activity tests, indicating that the configuration of the methylenecyclohexane ring could significantly influence the anti-inflammatory activity. These results suggest that structural variations could influence the biological activities of the compounds of this type and may warrant further studies in the future.

Furthermore, Western blotting was used to assess the changes in the protein expression levels of pro-inflammatory inducible nitric oxide synthase (iNOS) and cyclooxygenase 2 (COX-2) in a murine macrophage-like cell line, RAW264.7, stimulated with lipopolysaccharides (LPS). In the treatment of cells with 10 μM, briarenol E (**3**) reduced the levels of iNOS to 66.9%, in comparison with control cells stimulated with LPS only (Table 5 and Supplementary Figure S22). The results of the trypan blue exclusion test for cell viability showed that briaranes **1**–**3** did not induce significant cytotoxicity in RAW264.7 cells.

## 3. Experimental Section

### 3.1. General Experimental Procedures

Melting points of the natural products were determined using Fargo apparatus (Panchum Scientific, Kaohsiung, Taiwan), and the values were uncorrected. Optical rotation values were measured using a digital polarimeter (Jasco P-1010, Japan Spectroscopic Corp., Tokyo, Japan). IR spectra were obtained with a spectrophotometer (iS5 FT-IR, Thermo Scientific Nicolet, Waltham, MA, USA). NMR spectra were recorded on a NMR spectrometer (400 MHz Varian Mercury Plus, Varian, Palo Alto, CA, USA) using the residual CHCl_3_ signal (δ_H_ 7.26 ppm) and CDCl_3_ (δ_C_ 77.1 ppm) as the internal standard for ^1^H-NMR and ^13^C-NMR, respectively. Coupling constants (*J*) are presented in Hz. ESIMS and HRESIMS were recorded using a mass spectrometer (Bruker 7 Tesla solariX FTMS system, Bruker, Bremen, Germany). Column chromatography was carried out with 230–400 mesh silica gel (Merck, Darmstadt, Germany). TLC was performed on plates precoated with 0.25-mm-thick Kieselgel 60 F_254_ (Merck, Darmstadt, Germany); the plates were sprayed with 10% H_2_SO_4_ solution followed by heating to visualize the spots. Normal-phase HPLC (NP-HPLC) was performed using a HPLC system equipped with a pump (L-7110, Hitachi, Tokyo, Japan) and an injection port (7725, Rheodyne, Rohnert Park, CA, USA). A semi-preparative normal-phase LiChrospher 250 mm × 10 mm column (Hibar, Si 60, 5 μm; Merck Darmstadt, Germany) was used for HPLC. Reverse-phase HPLC (RP-HPLC) was performed using a system equipped with a pump (L-7100, Hitachi, Tokyo, Japan), a photodiode array detector (L-2455 Hitachi, Tokyo, Japan), an injection port (Rheodyne 7725) and a 250 mm × 21.2 mm column (Luna RP-18e, 5 μm, Torrance, CA, USA).

### 3.2. Animal Material

Specimens of *Briareum excavatum* were hand-picked by scuba divers in an area off the coast of Southern Taiwan in July 2011. The specimens were then stored in freezer immediately. A voucher specimen was deposited in the specimen bank of the National Museum of Marine Biology and Aquarium (NMMBA-TW-SC-2011-77) [13].

### 3.3. Extraction and Isolation

*B. excavatum* (wet weight, 6.32 kg; dry weight, 2.78 kg) samples were sliced and then extracted with a solvent mixture (methanol (MeOH):dichloromethane (DCM) = 1:1). The extract was partitioned between ethyl acetate (EtOAc) and H_2_O. The EtOAc layer was separated on silica gel followed by elution chromatography with a mixture of *n*-hexane/EtOAc (stepwise, 100:1, pure EtOAc) to yield 26 subfractions, A–Z. Fractions M, N, O, and P were combined and further separated on silica gel and eluted using *n*-hexane/EtOAc (stepwise, 4:1, pure EtOAc) to afford 30 subfractions, M1–M30. Fractions M8–M11 were combined and separated on silica gel followed by elution chromatography with a mixture of DCM/EtOAc (stepwise, 20:1, pure EtOAc) to yield 24 subfractions, M8A–M8X. Fraction M8K was separated on silica gel followed by elution chromatography with a solvent mixture (*n*-hexane:acetone = 3:1) to yield 11 subfractions, M8K1–M8K11. Fraction M8K5 was repurified by NP-HPLC, using a solvent mixture (*n*-hexane:acetone = 2:1) to afford **2** (2.3 mg). Fraction V was chromatographed on silica gel and eluted using a mixture of DCM/EtOAc (stepwise, 20:1, pure EtOAc) into 14 subfractions, V1–V14. Fraction V8 was separated by NP-HPLC using a mixture of DCM/EtOAc (1:1) as the mobile phase to afford **1** (2.0 mg). Fraction V7 was separated by NP-HPLC using a mixture of *n*-hexane/acetone (2:1) as the mobile phase to afford **3** (5.5 mg).

Briarenol C (**1**): white powder; m.p. 167–168 °C; [α]${}_{D}^{24}$ −5 (*c* 0.1, CHCl_3_); IR (neat) ν_max_ 3428, 1772, 1734 cm^−1^; ^1^H (400 MHz, CDCl_3_) and ^13^C (100 MHz, CDCl_3_) NMR data (see Table 1); ESIMS: *m/z* 545 [M + Na]^+^; HRESIMS: *m*/*z* 545.19930 (calcd. for C_26_H_34_O_11_ + Na, 545.19933).

Briarenol D (**2**): white powder; m.p. 158–159 °C; [α]${}_{D}^{24}$ +76 (*c* 0.1, CHCl_3_); IR (neat) ν_max_ 3445, 1777, 1733 cm^−1^; ^1^H (400 MHz, CDCl_3_) and ^13^C (100 MHz, CDCl_3_) NMR data (see Table 2); ESIMS: *m*/*z* 545 [M + Na]^+^; HRESIMS: *m*/*z* 545.19950 (calcd. for C_26_H_34_O_11_ + Na, 545.19933).

Briarenol E (**3**): white powder; m.p. 193–194 °C; [α]${}_{D}^{24}$ −32 (*c* 0.3, CHCl_3_); IR (neat) ν_max_ 3445, 1775, 1731 cm^−1^; ^1^H (400 MHz, CDCl_3_) and ^13^C (100 MHz, CDCl_3_) NMR data (see Table 3); ESIMS: *m/z* 539 [M + Na]^+^, 541 [M + 2 + Na]^+^; HRESIMS: *m/z* 539.16555 (calcd. for C_24_H_33_ClO_10_ + Na, 539.16545).

### 3.4. Generation of Superoxide Anions and Release of Elastase by Human Neutrophils

Human neutrophils were obtained by means of dextran sedimentation and Ficoll centrifugation. Measurements of superoxide anion generation and elastase release were carried out according to previously described procedures [14,15]. Briefly, superoxide anion production was assayed by monitoring the superoxide dismutase-inhabitable reduction of ferricytochrome c. Elastase release experiments were performed using MeO-Suc-Ala-Ala-Pro-Valp-nitroanilide as the elastase substrate.

### 3.5. In Vitro Anti-Inflammatory Assay

Murine macrophage-like cell line RAW264.7 was purchased from the American Type Culture Collection (ATCC, No TIB-71) (Manassas, VA, USA). The in vitro anti-inflammatory activities of compounds **1**–**3** were measured by investigating their inhibition effects on LPS-induced pro-inflammatory iNOS and COX-2 protein expressions in the macrophage cell line using western blot analysis [16,17,18]. Briefly, an inflammation response in RAW264.7 cells was induced by incubating cells in medium containing only LPS (10 ng/mL) without test compounds for 16 h. For the anti-inflammatory activity assay, Compounds **1**–**3** or dexamethasone (10 μM) were added to the cells 10 min before LPS treatment. After incubation, the cells were lysed and the protein lysates analyzed by Western blotting. The protein expression levels were determined based on the immunoreactivity of proteins to antibodies, and were calculated with respect to the average optical density of the corresponding LPS-stimulated cells. Moreover, the effects of Compounds **1**–**3** on the viability of RAW 264.7 cells were also evaluated by the trypan blue exclusion test [17,18]. For statistical analysis, the data were analyzed by one-way analysis of variance (ANOVA), followed by the Student–Newman–Keuls *post hoc* test for multiple comparisons. A significant difference was defined as a *p*-value of <0.05.

## 4. Conclusions

The octocoral *Briareum excavatum* has proven to be a rich source of interesting briarane-related natural products with complex structures and extensive bioactivities. It is interesting to note that briarane-related natural products isolated from *B. excavatum* possessing a twisted boat conformation are rarely found. Briarenol D (**2**) is a compound potentially suitable for future development. This interesting species has been transplanted to culture tanks located in the National Museum of Marine Biology and Aquarium. A large quantity of cultured *B. excavatum* is being cultivated for extraction of natural material in order to establish a stable supply of bioactive substances.

## Figures and Tables

**Table 1:** ^1^H (400 MHz, CDCl_3_) and ^13^C (100 MHz, CDCl_3_) NMR data and ^1^H-^1^H COSY and HMBC correlations for briarane **1**.

Position	δ_H_ (*J* in Hz)	δ_C_, Multiple	^1^H-^1^H COSY	HMBC
1		47.1, C		
2	4.80 d (4.4)	72.0, CH	H_2_-3	C-1, -4, -15, acetate carbonyl
3α/β	1.96 m; 3.15 dd (12.8, 11.6)	37.7, CH_2_	H-2, H-4	C-1, -2, -4, -5
4	5.19 dd (12.8, 6.0)	73.0, CH	H_2_-3	C-3, -6, -16
5		144.8, C		
6	5.59 d (10.0)	123.4, CH	H-7, H_3_-16	C-8
7	5.94 d (10.0)	73.2, CH	H-6	C-5
8		71.4, C		
9	3.95 br s	72.3, CH	H-10	C-17
10	3.39 d (5.2)	44.4, CH	H-9	C-1, -2, -9, -11, -12, -15, -20
11		150.6, C		
12	4.51 dd (8.8, 7.6)	65.5, CH	H_2_-13	n.o. ^a^
13α/β	1.55 m; 2.62 ddd (16.0, 8.8, 4.4)	37.2, CH_2_	H-12, H-14	C-1, -11, -12, -14
14	4.74 d (4.4)	74.1, CH	H_2_-13	C-1, -10, -12, acetate carbonyl
15	1.34 s	16.4, CH_3_		C-1, -2, -10, -14
16	2.20 d (1.2)	25.6, CH_3_	H-6	C-4, -5, -6
17		60.6, C		
18	1.53 s	9.5, CH_3_		C-8, -17, -19
19		171.9, C		
20a	5.13 d (1.2)	109.3, CH_2_	H-20b	C-10, -12
b	5.03 s		H-20a	C-10, -12
OAc-2		170.6, C		
	2.01 s	20.9, CH_3_		Acetate carbonyl
OAc-4		170.3, C		
	1.91 s	21.1, CH_3_		Acetate carbonyl
OAc-14		170.3, C		
	2.04 s	21.0, CH_3_		Acetate carbonyl

^a^ n.o. = not observed.

**Table 2:** ^1^H (400 MHz, CDCl_3_) and ^13^C (100 MHz, CDCl_3_) NMR data and ^1^H-^1^H COSY and HMBC correlations for briarane **2**.

Position	δ_H_ (*J* in Hz)	δ_C_, Multiple	^1^H-^1^H COSY	HMBC
1		48.0, C		
2	5.01 d (7.2)	72.5, CH	H_2_-3	C-1, -3, -4, -10, -15, acetate carbonyl
3α/β	2.00 m; 3.07 dd (15.2, 12.4)	37.6, CH_2_	H-2, H-4	C-1, -2, -4, -5
4	5.08 dd (12.4, 5.6)	72.8, CH	H_2_-3	C-3, -5, -6, -16, acetate carbonyl
5		143.6, C		
6	5.47 ddd (9.6, 1.2, 1.2)	123.4, CH	H-7, H_3_-16	C-4, -16
7	5.91 d (9.6)	74.2, CH	H-6	C-5, -6, -19
8		71.2, C		
9	4.35 br s	73.4, CH	H-10, OH-9	C-8, -10, -11
10	2.96 d (2.8)	44.1, CH	H-9	C-1, -2, -8, -9, -11, -12, -14, -15, -20
11		151.5, C		
12	4.31 dd (6.8, 6.8)	69.7, CH	H_2_-13	C-10, -11, -13, -14, -20
13α/β	2.19 ddd (15.2, 6.8, 3.2); 1.81 ddd (15.2, 6.8, 3.6)	36.6, CH_2_	H-12, H-14	C-1, -11, -12, -14
14	4.76 dd (3.6, 3.2)	73.8, CH	H_2_-13	C-10, -12
15	1.30 s	14.7, CH_3_		C-1, -2, -10, -14
16	2.11 d (1.2)	25.4, CH_3_	H-6	C-4, -5, -6
17		62.2, C		
18	1.52 s	10.0, CH_3_		C-8, -17, -19
19		172.0, C		
20a	5.29 s	110.9, CH_2_	H-20b	C-10, -11, -12
b	5.07 s		H-20a	C-10, -11, -12
OAc-2		170.4, C		
	2.01 s	21.0, CH_3_		Acetate carbonyl
OAc-4		170.3, C		
	2.04 s	21.0, CH_3_		Acetate carbonyl
OAc-14		170.6, C		
	1.96 s	21.3, CH_3_		Acetate carbonyl
OH-9	3.00 d (4.8)		H-9	

**Table 3:** ^1^H (400 MHz, CDCl_3_) and ^13^C (100 MHz, CDCl_3_) NMR data and ^1^H–^1^H COSY and HMBC correlations for briarane **3**.

Position	δ_H_ (*J* in Hz)	δ_C_, Multiple	^1^H-^1^H COSY	HMBC
1		45.3, C		
2	4.99 d (6.4)	73.9, CH	H_2_-3	C-1, -3, -10, -14, -15, acetate carbonyl
3α/β	1.34 dd (15.6, 4.8); 3.37 ddd (15.6, 12.8, 6.4)	35.8, CH_2_	H-2, H-4	C-1, -2, -4, -5
4	4.80 dd (12.8, 4.8)	76.3, CH	H_2_-3	C-2, -3, -6, -8, -16
5		138.0, C		
6	5.51 ddd (2.8, 2.4, 2.4)	55.1, CH	H-7, H_2_-16	C-5, -16
7	4.74 d (2.8)	80.5, CH	H-6	C-5, -6
8		82.0, C		
9	4.87 d (3.6)	76.3, CH	OH-9	C-1, -8, -10, -11, -17
10	2.16 s	40.4, CH	n.o. ^a^	C-1, -2, -8, -9, -11, -12, -14, -15
11		78.4, C		
12	3.49 m	76.2, CH	H_2_-13, OH-12	n.o.
13α/β	1.96 ddd (15.6, 3.6, 2.8); 2.43 ddd (15.6, 4.0, 2.8)	28.0, CH_2_	H-12, H-14	C-1, -12, -14
14	5.18 dd (2.8, 2.8)	76.4, CH	H_2_-13	C-2, -10, -12, acetate carbonyl
15	1.53 s	16.6, CH_3_		C-1, -2, -10, -14
16a	5.29 d (2.4)	115.7, CH_2_	H-6, H-16b	C-4, -6
b	5.46 d (2.4)		H-6, H-16a	C-4, -5, -6
17	2.59 q (7.2)	50.2, CH	H_3_-18	C-8, -9, -18, -19
18	1.29 d (7.2)	8.2, CH_3_	H-17	C-8, -17, -19
19		175.9, C		
20	1.54 s	29.5, CH_3_		C-10, -11, -12
OAc-2		170.8, C		
	1.99 s	21.2, CH_3_		Acetate carbonyl
OAc-14		169.2, C		
	2.04 s	21.1, CH_3_		Acetate carbonyl
OH-9	2.78 d (3.6)		H-9	C-8, -9
OH-12	2.71 d (9.2)		H-12	

^a^ n.o. = not observed.

**Table 4:** Inhibitory effects of briaranes **1**–**3** on superoxide anion generation and elastase release by human neutrophils in response to fMet-Leu-Phe/Cytochalastin B.

Compound	Superoxide Anions	Elastase Release
	IC_50_ (μM) ^a^	IC_50_ (μM)
**1**	>10	>10
**2**	>10	4.65 ± 1.50
**3**	>10	>10
**LY294002** ^b^	1.39 ± 0.32	3.30 ± 0.11

^a^ Concentration necessary for 50% inhibition (IC_50_); results are presented as mean ± S. E. M. (*n* = 3). ^b^ LY294002 (2-morpholin-4-yl-8-phenylchromen-4-one) was used as reference compound.

**Table 5:** Effects of briaranes **1**–**3** on LPS-induced iNOS and COX-2 protein expression in macrophages.

Compound	iNOS	COX-2
	Expression (% of LPS Group)	Expression (% of LPS Group)
Control	0.79 ± 0.01	1.00 ± 0.02
LPS	100.00 ± 7.48	100.00 ± 18.39
1	93.22 ± 22.59	100.41 ± 1.08
2	78.35 ± 0.73	94.28 ± 21.35
3	66.86 ± 3.86	119.42 ± 1.33
DEX ^a^	56.18 ± 4.53	17.42 ± 2.53

^a^ Dexamethasone (DEX, 10 μM) was used as a positive control.

## Supplementary Materials

HRESIMS, 1D, 2D NMR spectra, and figure of Western blot of new compounds **1**–**3** are available online.
