# Supplementary material for: Briarenols C–E, New Polyoxygenated Briaranes from the Octocoral Briareum excavatum
Source: Molecules. 2017 Mar 17;22(3):475. doi: 10.3390/molecules22030475 (PMC6155408; doi:10.3390/molecules22030475)
Supplement: Supplementary file 1 [file molecules-22-00475-s001.pdf]

Supporting Information

Briarenols C–E, New Polyoxygenated Briaranes from the Octocoral *Briareum excavatum*

Yin-Di Su, Nan-Fu Chen, Tsong-Long Hwang, Zuo-Jian Liao, Kuan-Hao Tsui, Zhi-Hong Wen, Yang-Chang Wu and Ping-Jyun Sung

No	Content	page
S1.	HRESIMS spectrum of compound **1**.	2
S2.	^{1}H NMR spectrum (400 MHz) of compound **1** in $CDCl_3$.	2
S3.	^{13}C NMR spectrum (100 MHz) of compound **1** in $CDCl_3$.	3
S4.	gHSQC spectrum (400 MHz) of compound **1** in $CDCl_3$.	3
S5.	^{1}H–^{1}H COSY spectrum (400 MHz) of compound **1** in $CDCl_3$.	4
S6.	gHMBC spectrum (400 MHz) of compound **1** in $CDCl_3$.	4
S7.	NOESY spectrum (400 MHz) of compound **1** in $CDCl_3$.	5
S8.	HRESIMS spectrum of compound **2**.	5
S9.	^{1}H NMR spectrum (400 MHz) of compound **2** in $CDCl_3$.	6
S10.	^{13}C NMR spectrum (100 MHz) of compound **2** in $CDCl_3$.	6
S11.	gHSQC spectrum (400 MHz) of compound **2** in $CDCl_3$.	7
S12.	^{1}H–^{1}H COSY spectrum (400 MHz) of compound **2** in $CDCl_3$.	7
S13.	gHMBC spectrum (400 MHz) of compound **2** in $CDCl_3$.	8
S14.	NOESY spectrum (400 MHz) of compound **2** in $CDCl_3$.	8
S15.	HRESIMS spectrum of compound **3**.	9
S16.	^{1}H NMR spectrum (400 MHz) of compound **3** in $CDCl_3$.	9
S17.	^{13}C NMR spectrum (100 MHz) of compound **3** in $CDCl_3$.	10
S18.	gHSQC spectrum (400 MHz) of compound **3** in $CDCl_3$.	10
S19.	^{1}H–^{1}H COSY spectrum (400 MHz) of compound **3** in $CDCl_3$.	11
S20.	gHMBC spectrum (400 MHz) of compound **3** in $CDCl_3$.	11
S21.	NOESY spectrum (400 MHz) of compound **3** in $CDCl_3$.	12
S22.	Effects of briaranes **1–3** on LPS-induced iNOS and COX-2 protein expression in macrophages.	13

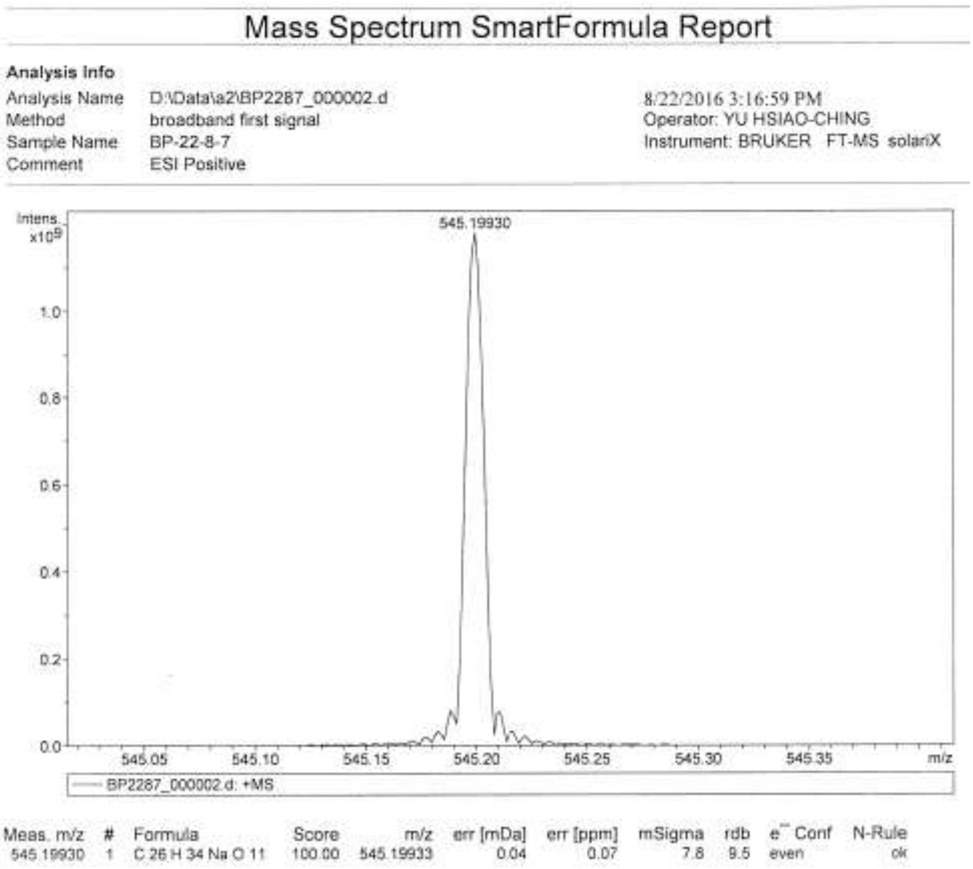

S1. HRESIMS spectrum of compound **1**.

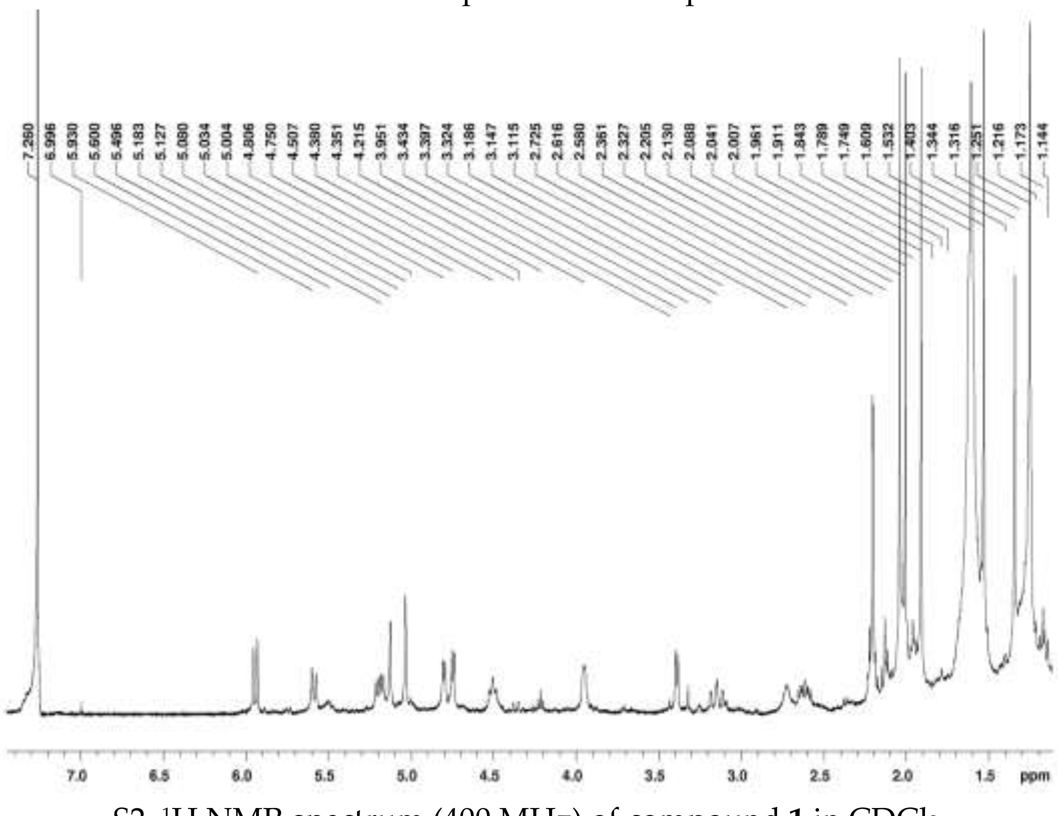

S2. ^{1}H NMR spectrum (400 MHz) of compound **1** in $CDCl_3$.

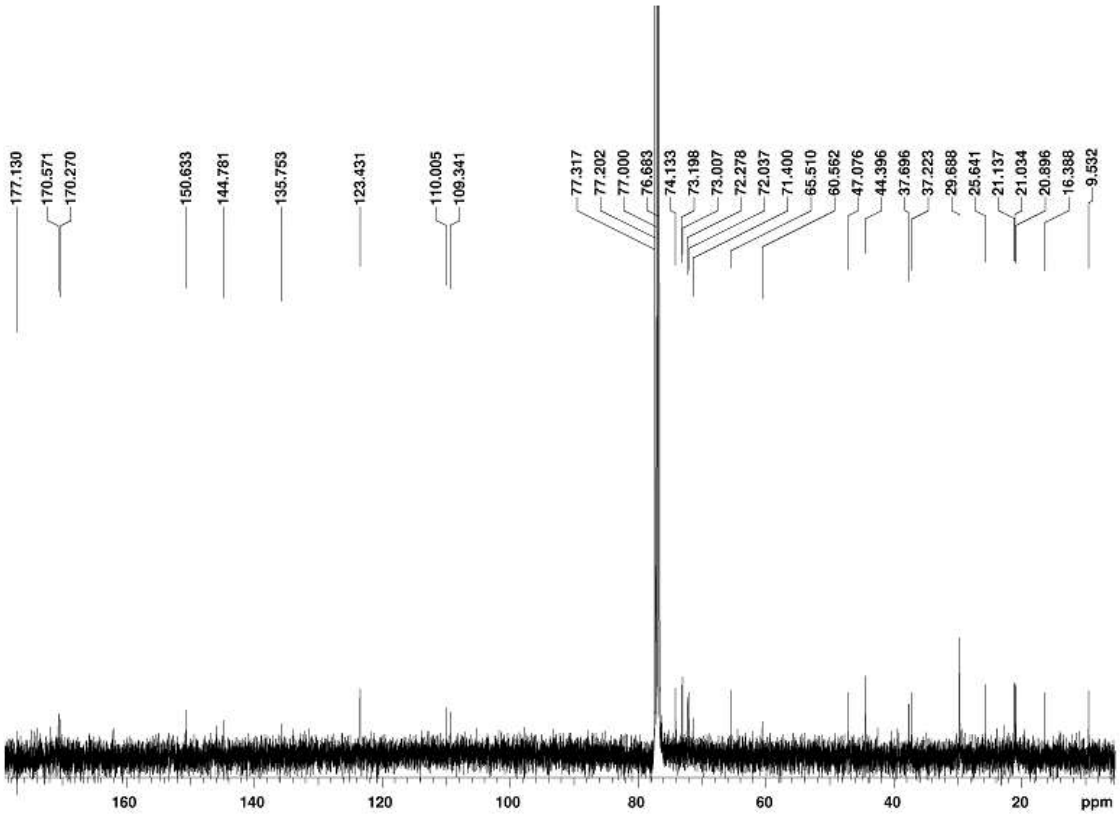

S3. ^{13}C NMR spectrum (100 MHz) of compound **1** in $CDCl_3$.

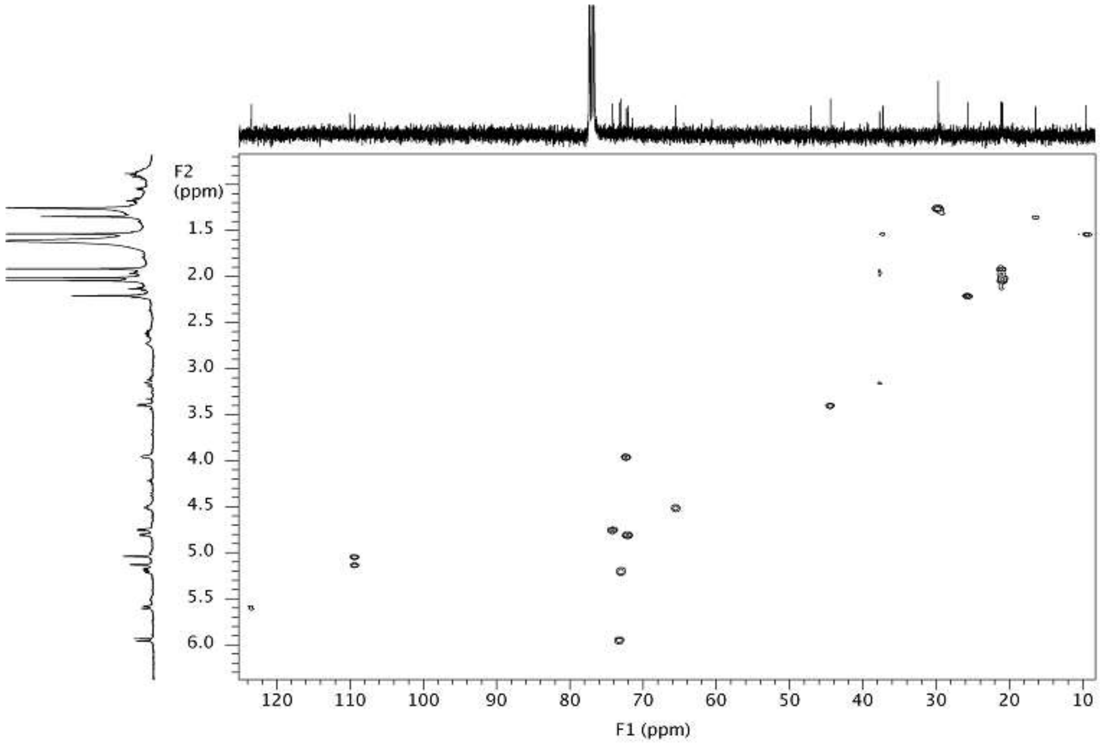

S4. gHSQC spectrum (400 MHz) of compound **1** in $CDCl_3$.

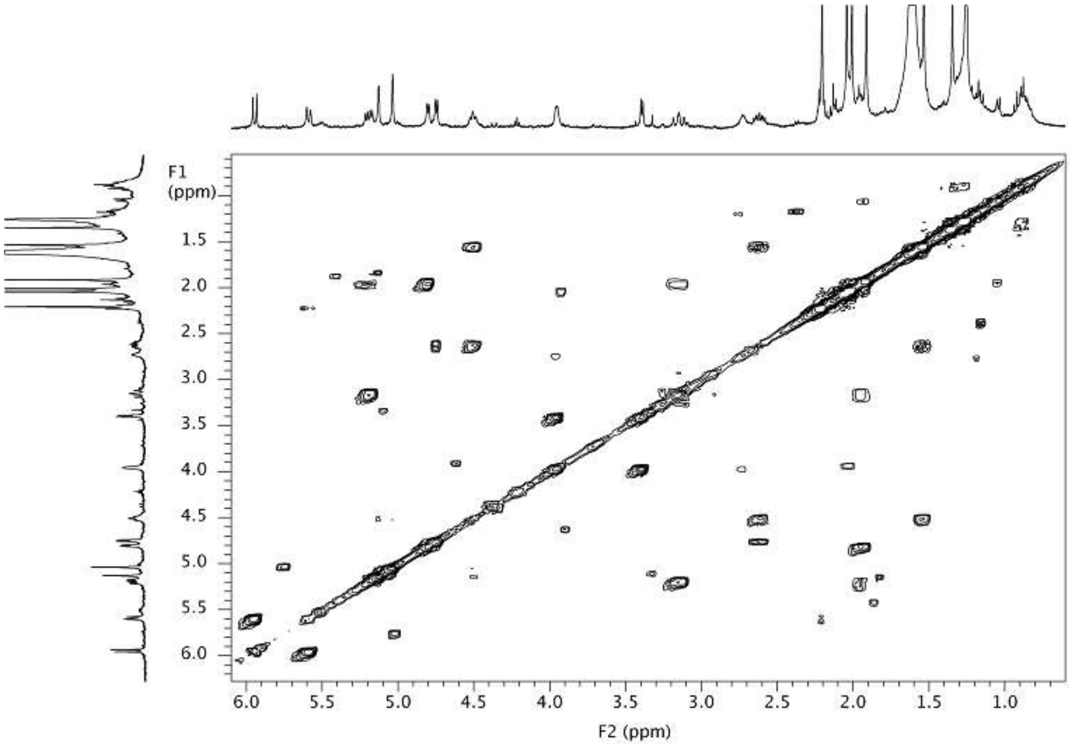

S5. ^{1}H–^{1}H COSY spectrum (400 MHz) of compound **1** in $CDCl_3$.

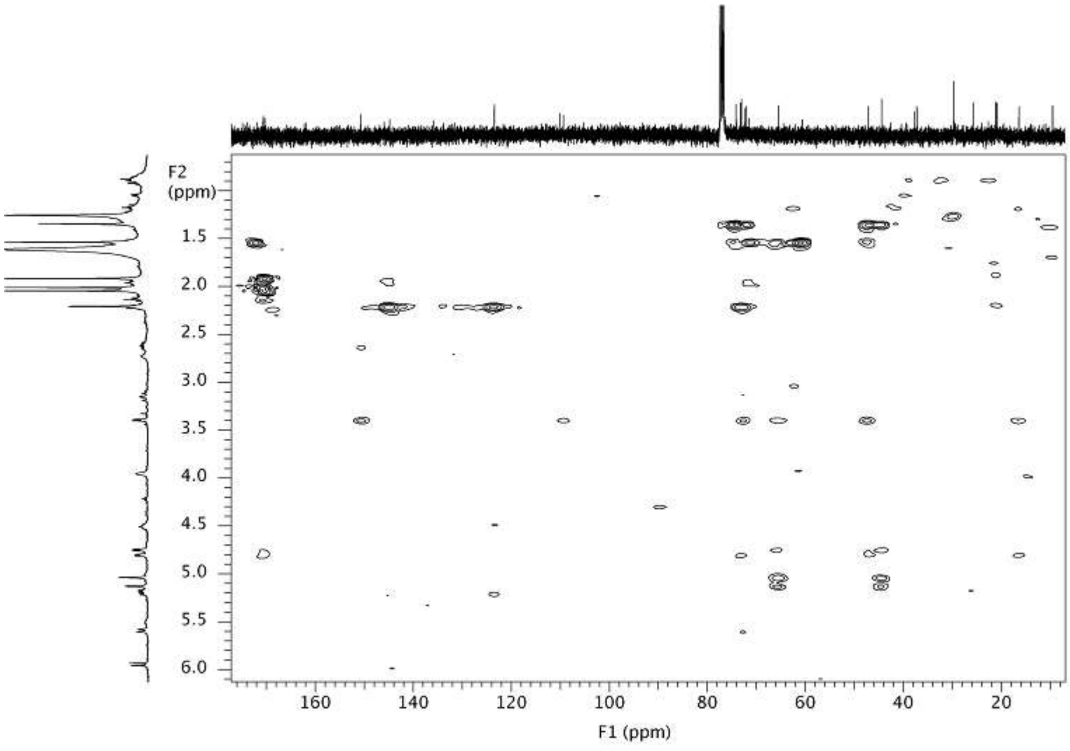

S6. gHMBC spectrum (400 MHz) of compound **1** in $CDCl_3$.

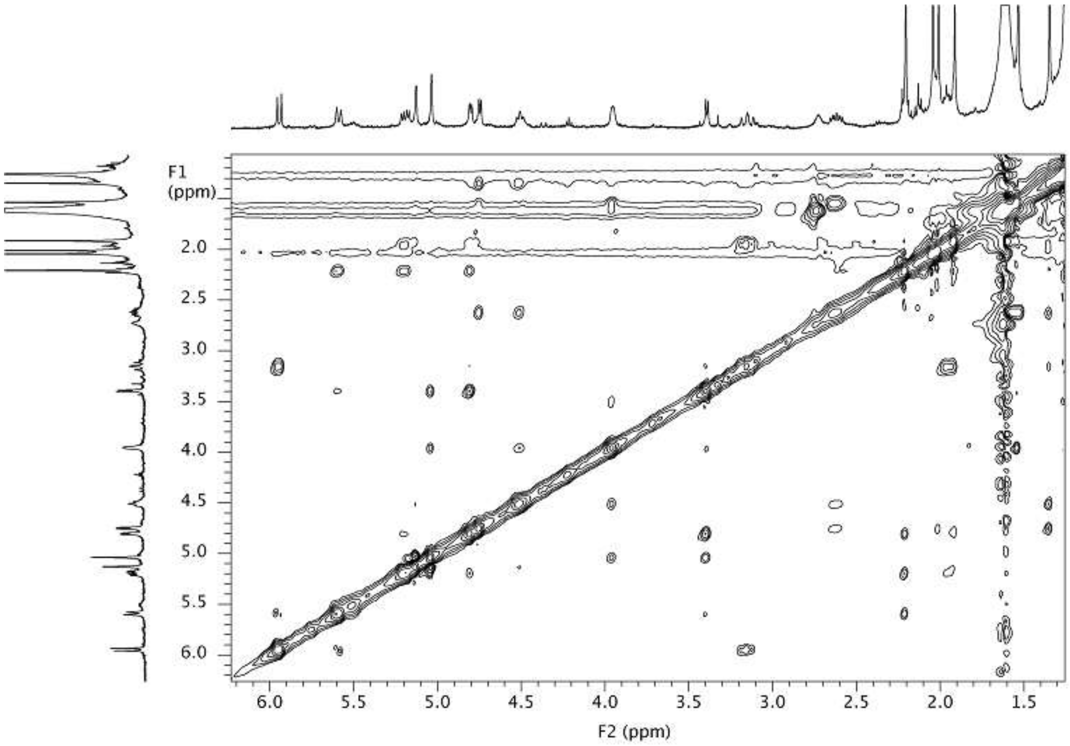

S7. NOESY spectrum (400 MHz) of compound **1** in $CDCl_3$.

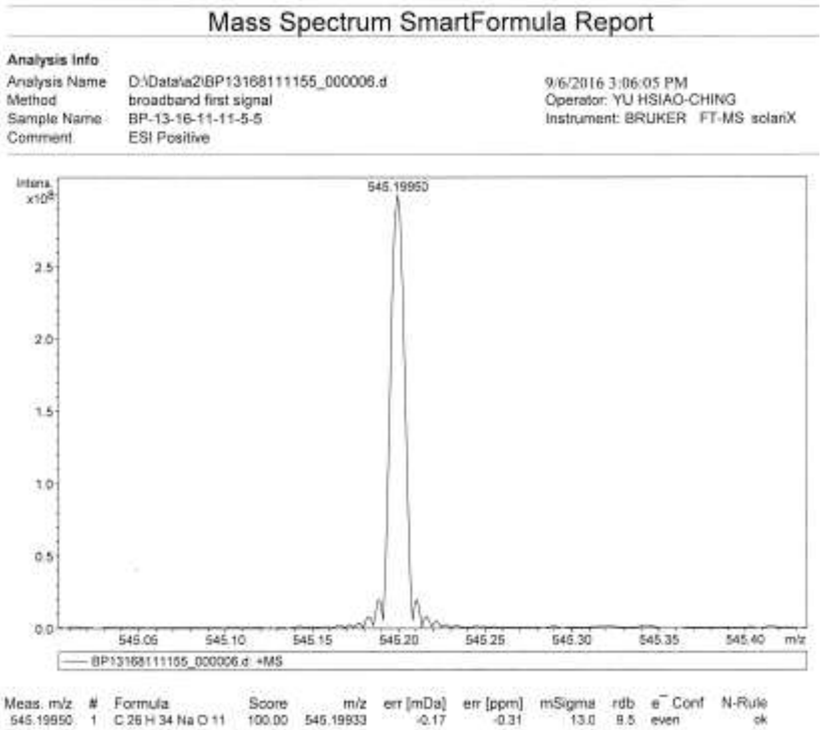

S8. HRESIMS spectrum of compound **2**.

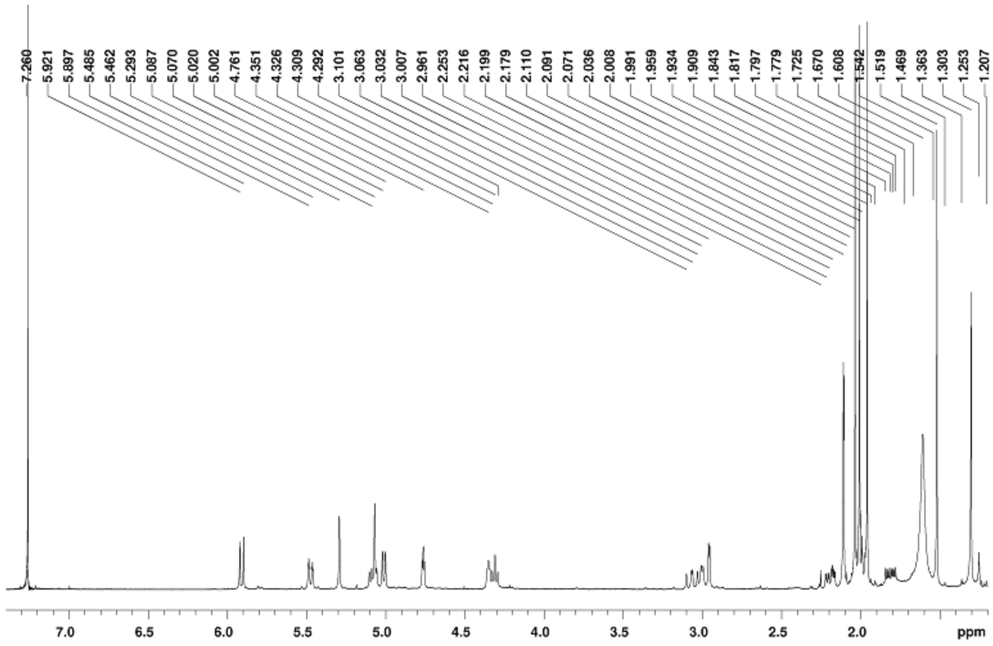

S9. ^{1}H NMR spectrum (400 MHz) of compound **2** in $CDCl_3$.

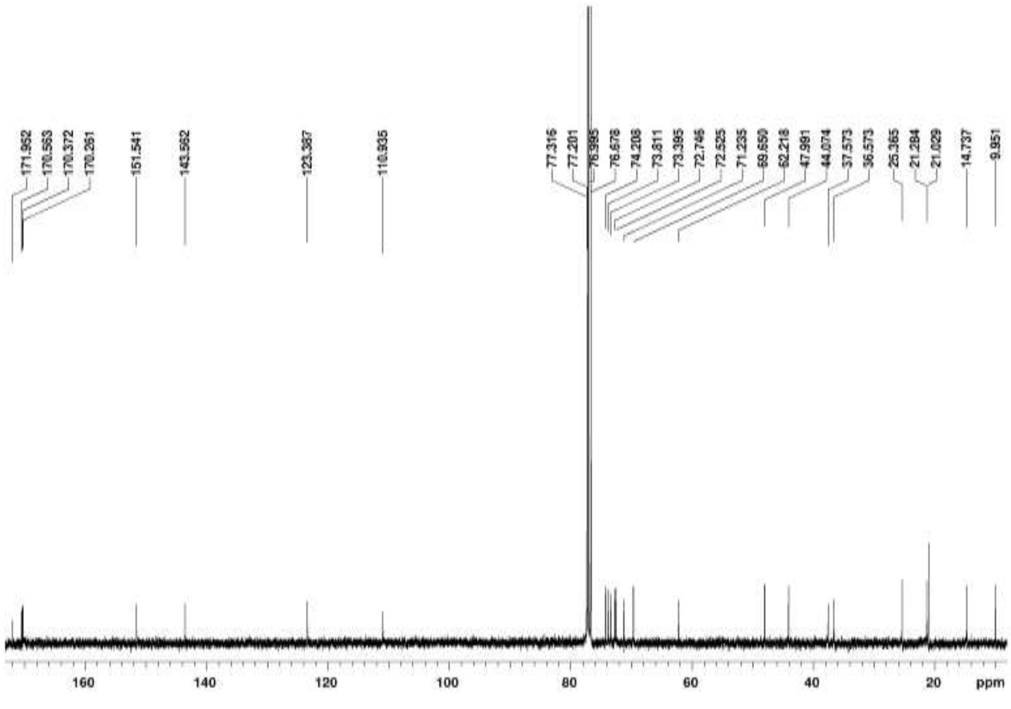

S10. ^{13}C NMR spectrum (100 MHz) of compound **2** in $CDCl_3$.

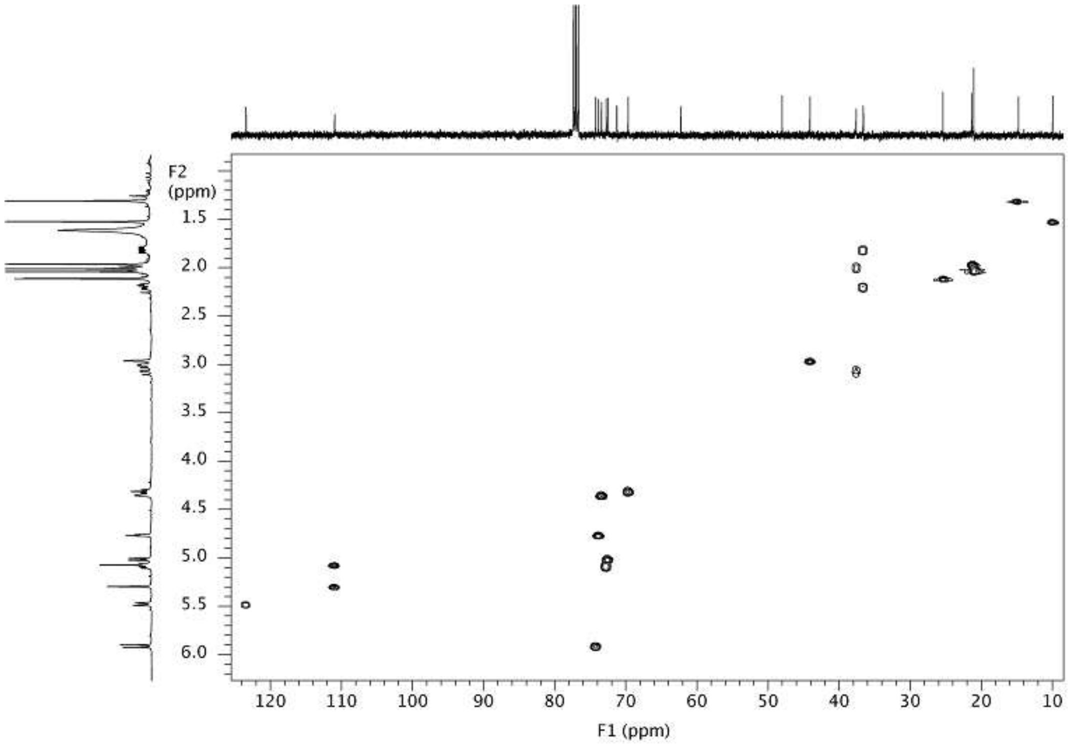

S11. gHSQC spectrum (400 MHz) of compound **2** in $CDCl_3$.

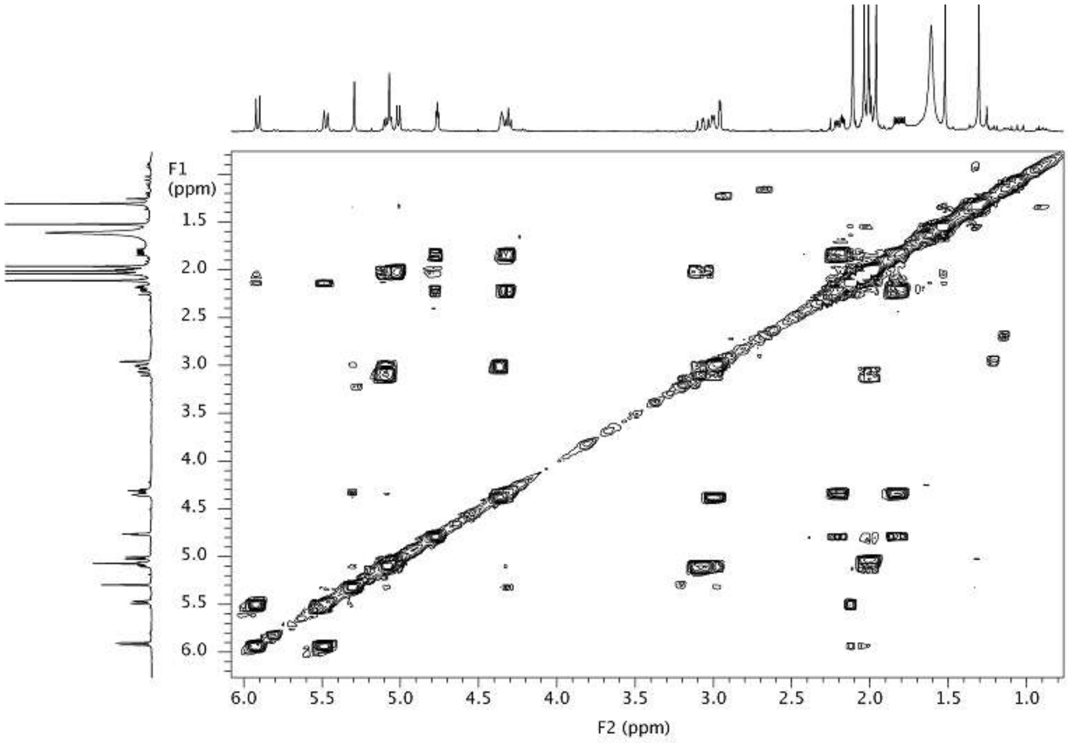

S12. ^{1}H–^{1}H COSY spectrum (400 MHz) of compound **2** in $CDCl_3$.

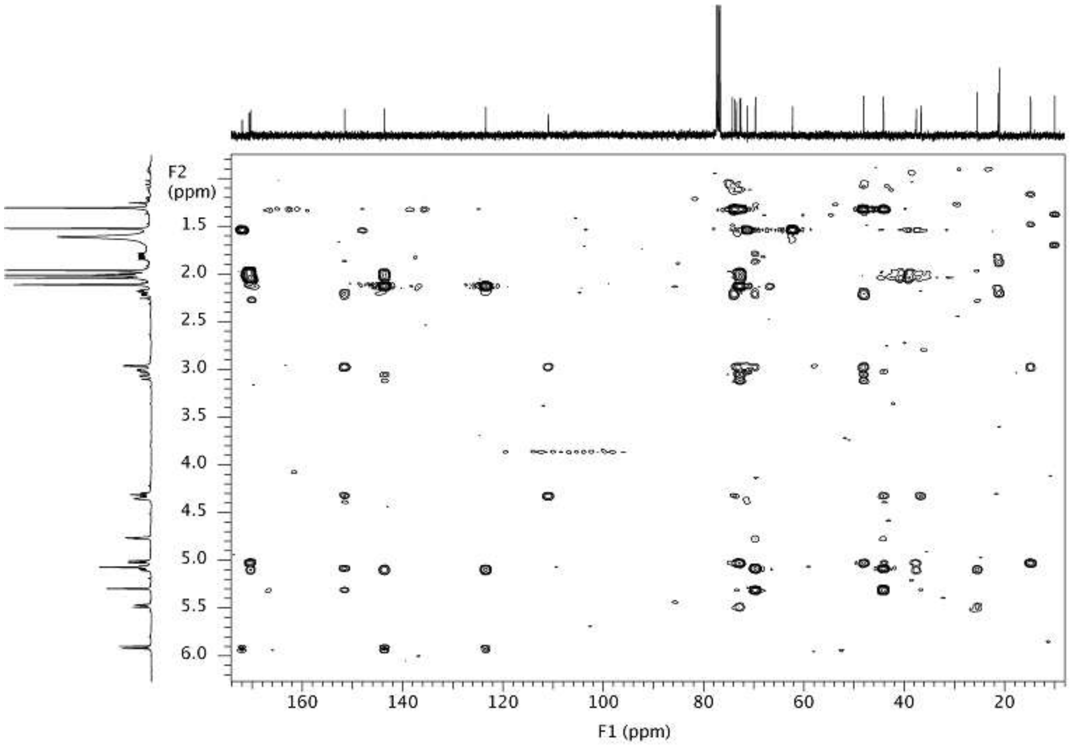

S13. gHMBC spectrum (400 MHz) of compound **2** in $CDCl_3$.

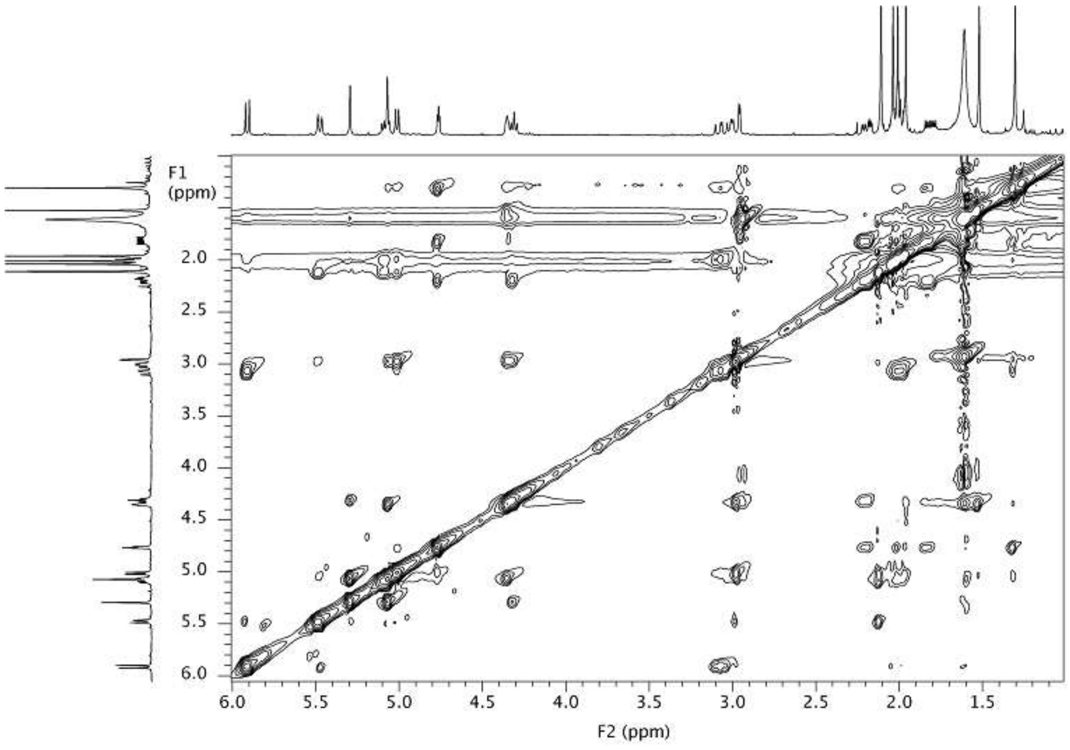

S14. NOESY spectrum (400 MHz) of compound **2** in $CDCl_3$.

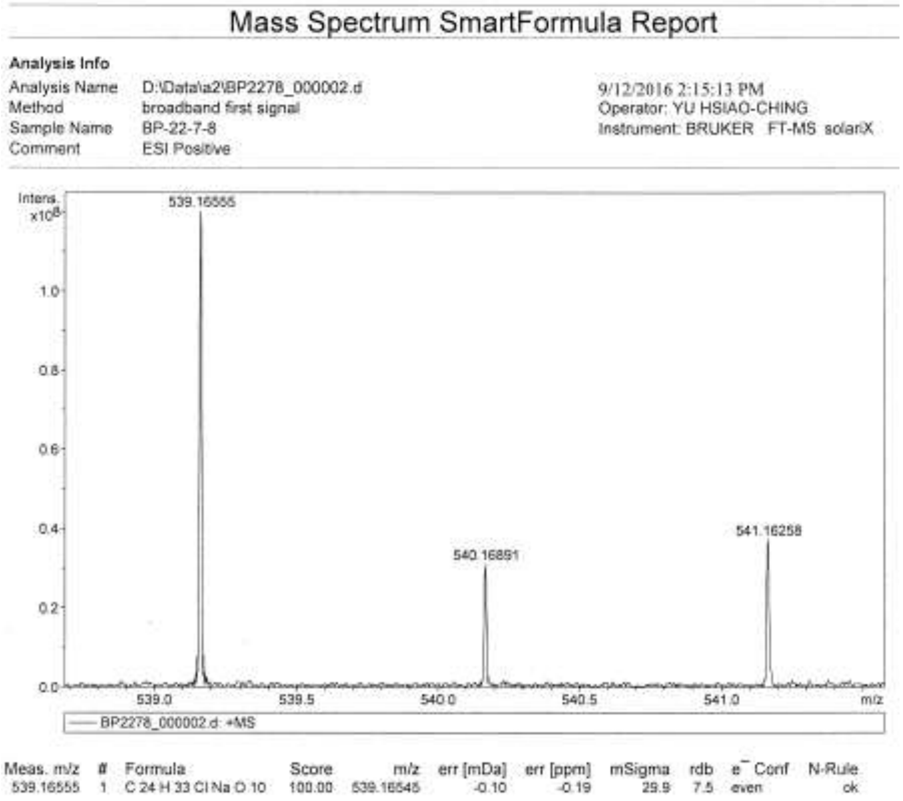

S15. HRESIMS spectrum of compound **3**.

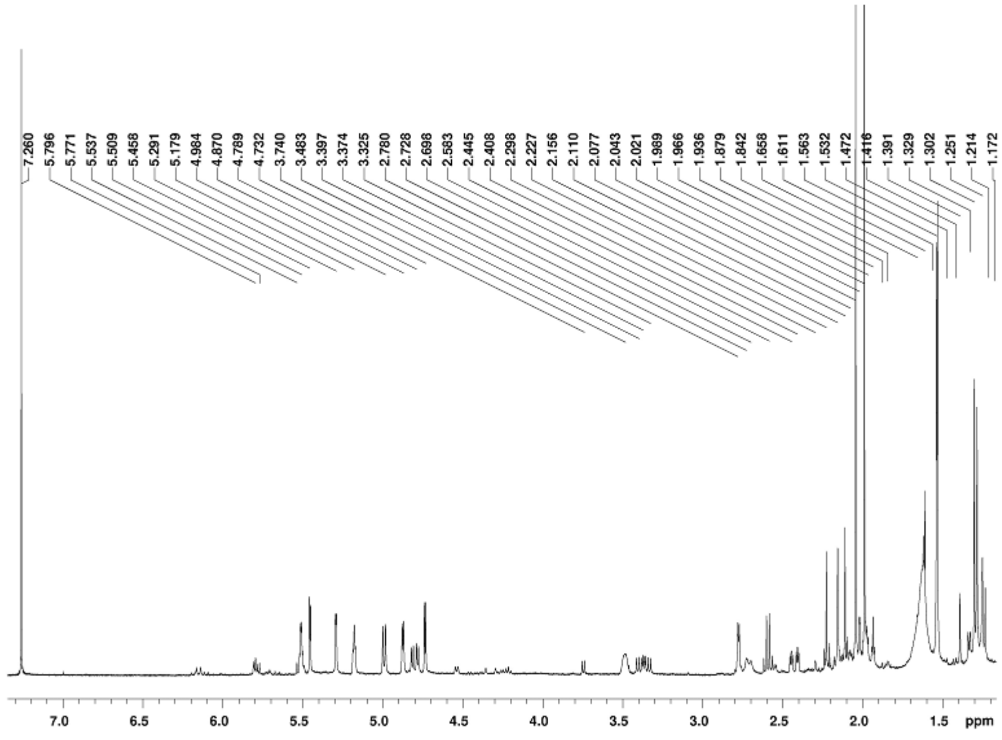

S16. ^{1}H NMR spectrum (400 MHz) of compound **3** in $CDCl_3$.

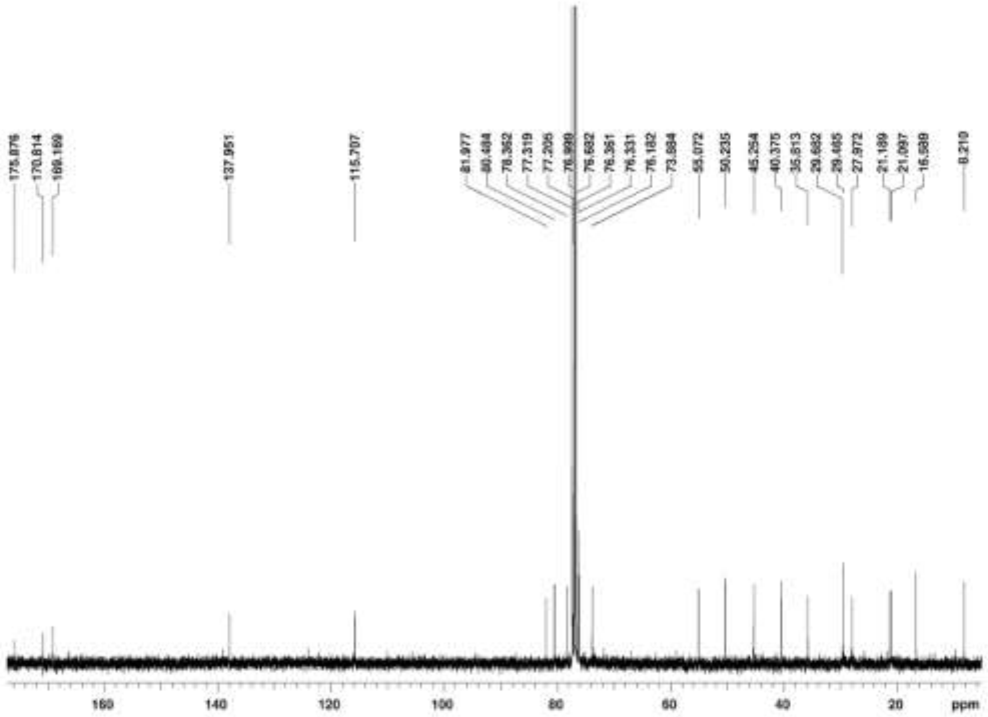

S17. ^{13}C NMR spectrum (100 MHz) of compound **3** in $CDCl_3$.

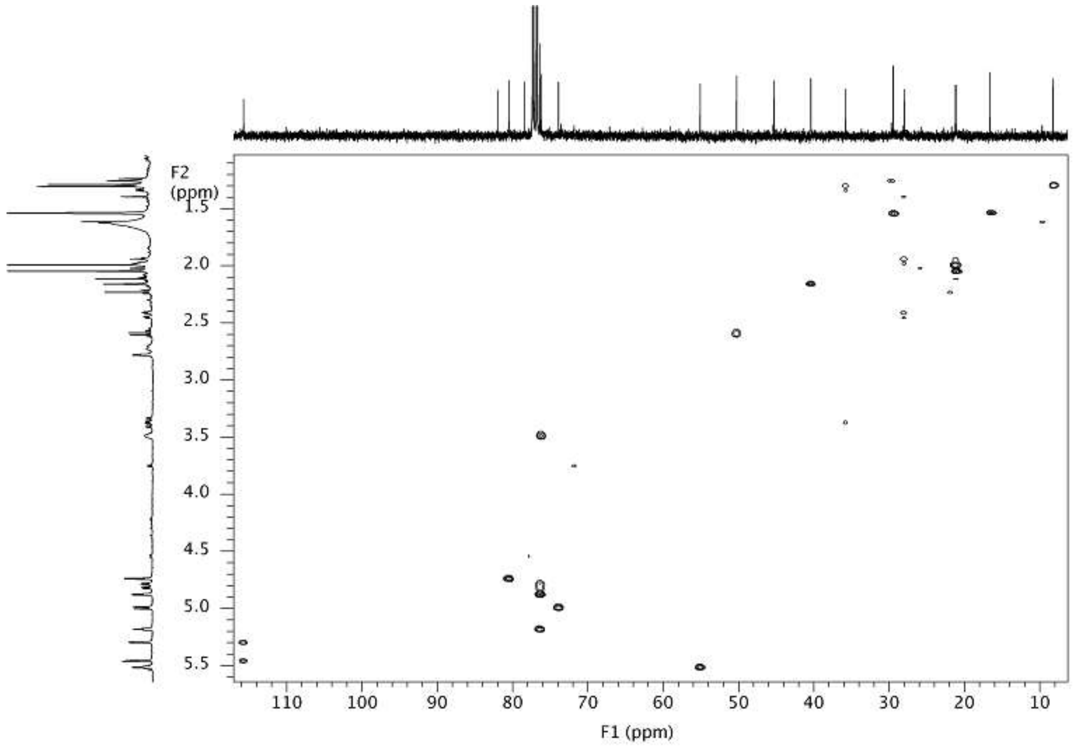

S18. gHSQC spectrum (400 MHz) of compound **3** in $CDCl_3$.

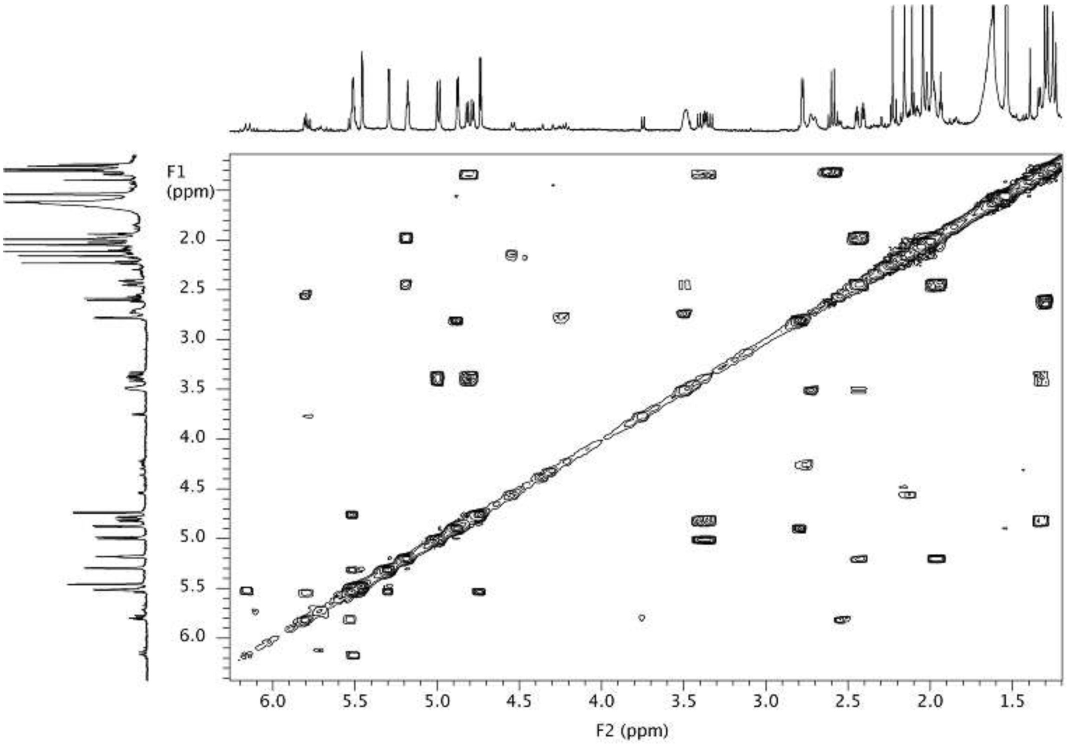

S19. ^1H–^1H COSY spectrum (400 MHz) of compound **3** in $CDCl_3$.

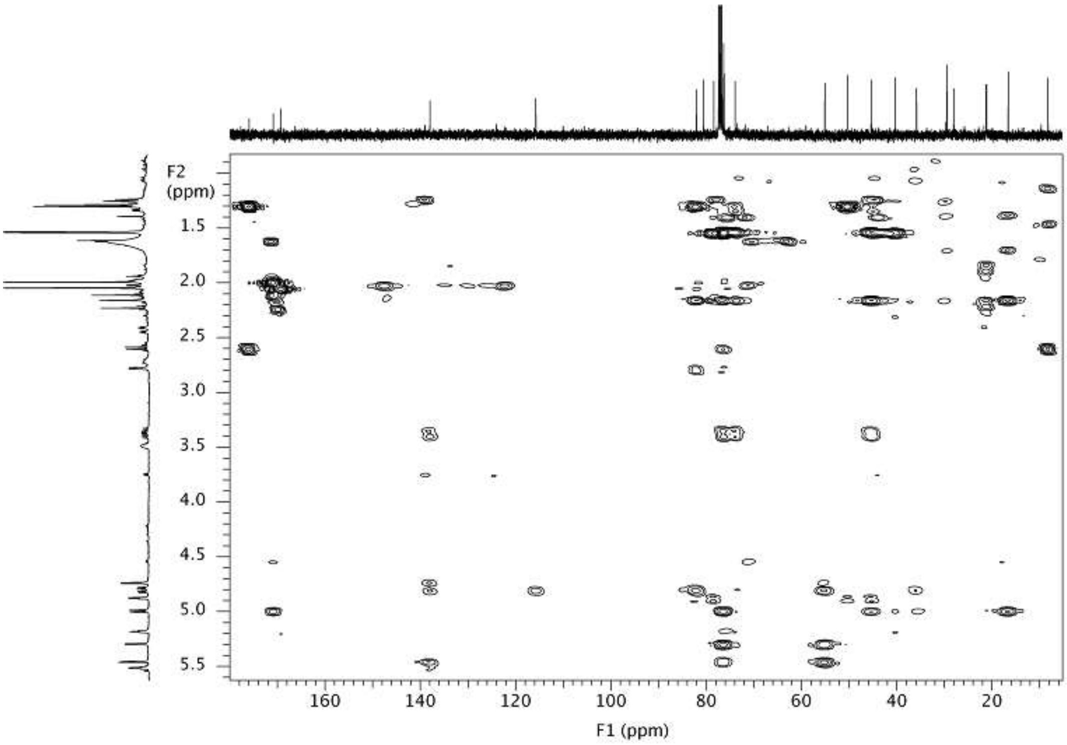

S20. gHMBC spectrum (400 MHz) of compound **3** in $CDCl_3$.

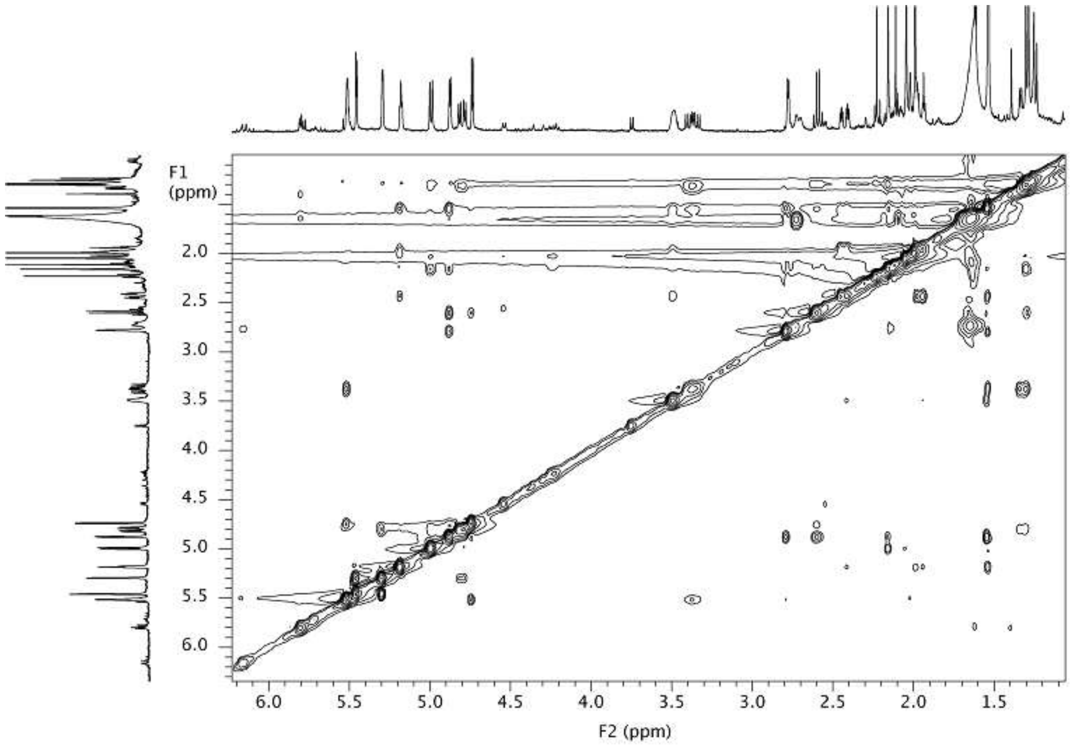

S21. NOESY spectrum (400 MHz) of compound **3** in $CDCl_3$.

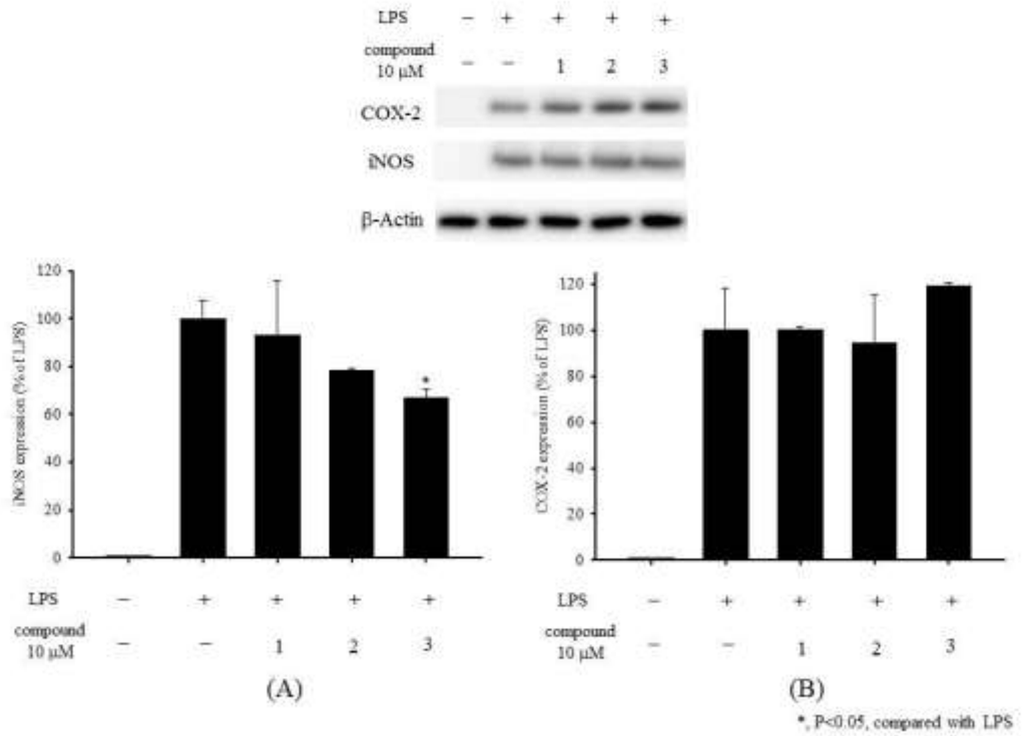

S22. Effects of compounds briarenols C–E (**1**–**3**) on pro-inflammatory iNOS and COX-2 protein expression in the LPS-stimulated murine macrophage cell line RAW264.7. (**A**) The relative density of iNOS immunoblot; (**B**) the relative density of COX-2 immunoblot. The relative intensity of the LPS-stimulated group was taken to be 100%. Band intensities were quantified by densitometry and are indicated as the percent change relative to that of the LPS-stimulated group. briarenols C–E (**1**–**3**) significantly inhibited LPS-induced iNOS and COX-2 protein expression in macrophages. The experiments were repeated three times (* $p < 0.05$, significantly different from the LPS-stimulated group).
